# Validity, reliability, and responsiveness of a self-reported foot and ankle score (SEFAS)

**DOI:** 10.3109/17453674.2012.657579

**Published:** 2012-04-24

**Authors:** Maria Cöster, Magnus K Karlsson, Jan-Åke Nilsson, Åke Carlsson

**Affiliations:** ^1^Department of Orthopedics, Kalmar Hospital, Kalmar; ^2^Department of Clinical Sciences and Orthopaedics, Lund University, Skåne University Hospital in Malmö, Malmö, Sweden

## Abstract

**Background and purpose:**

A questionnaire was introduced by the New Zealand Arthroplasty Registry for use when evaluating the outcome of total ankle replacement surgery. We evaluated the reliability, validity, and responsiveness of the modified Swedish version of the questionnaire (SEFAS) in patients with osteoarthritis or inflammatory arthritis before and/or after their ankle was replaced or fused.

**Patients and methods:**

The questionnaire was translated into Swedish and cross-culturally adapted according to a standardized procedure. It was sent to 135 patients with ankle arthritis who were scheduled for or had undergone surgery, together with the foot and ankle outcome score (FAOS), the short form 36 (SF-36) score, and the EuroQol (EQ-5D) score. Construct validity was evaluated with Spearman’s correlation coefficient when comparing SEFAS with FAOS, SF-36, and EQ-5D, content validity by calculating floor and ceiling effects, test-retest reliability with intraclass correlation coefficient (ICC), internal consistency with Cronbach’s alpha (n = 62), agreement by Bland-Altman plot, and responsiveness by effect size and standardized response mean (n = 37).

**Results:**

For construct validity, we correlated SEFAS with the other scores and 70% or more of our predefined hypotheses concerning correlations could be confirmed. There were no floor or ceiling effects. ICC was 0.92 (CI 95%: 0.88–0.95), Cronbach’s alpha 0.96, effect size was 1.44, and the standardized response mean was 1.00.

**Interpretation:**

SEFAS is a self-reported foot and ankle score with good validity, reliability and responsiveness, indicating that the score can be used to evaluate patients with osteoarthritis or inflammatory arthritis of the ankle and outcome of surgery.

A self-administered ankle questionnaire based on the validated Oxford-12 questionnaire for total hip replacement has been constructed by the New Zealand National Joint Registry. The aim was to collect patient-based data after total ankle replacement (TAR) as an amendment to medically recorded joint-specific data and it proved to be useful, particularly in the prediction of failures (Hosman et al. 2007). However, the original version of the questionnaire has not been validated.

Already existing self-administrated foot and ankle scores contain numerous questions and can be complicated to use. For osteoarthritis and inflammatory arthritis of the ankle, there are few validated instruments and they are seldom used (Budiman-Mak et al. 1991, Button and Pinney 2004, Naal et al. 2010). None can be regarded as the gold standard. The generic, self-administered questionnaires short form 36 (SF-36) (Sullivan et al. 1995, Patel et al. 2007) and EuroQol (EQ-5D) (EuroQol Group 1990) are useful when evaluating general health, but they may be less effective when evaluating joint-specific disability.

Thus, there is a need for a simple, self-administered and ankle-specific score that is capable of evaluating pain and functional status in patients with osteoarthritis and inflammatory arthritis of the ankle, and the outcome of surgical interventions—not least when collecting data for national surgical registers. We therefore assessed the validity, the reliability, and the responsiveness of the modified Swedish version of the New Zealand total ankle replacement questionnaire, here called the self-reported foot and ankle score (SEFAS), in relation to 3 established self-administered scoring systems. The reason for choosing the foot and ankle outcome score (FAOS) for comparison was that this region-specific score is the only one available in Swedish and the reason for choosing the generic scores SF-36 and EQ-5D was because they are widely used.

## Patients and methods

### The self-reported foot and ankle score (SEFAS)

SEFAS is based on the New Zealand total ankle questionnaire (Hosman et al. 2007) that was originally derived from the validated Oxford-12 hip questionnaire (Dawson et al. 1996). 8 of the 12 questions are the same as in the original questionnaire, while 4 of the questions have been replaced with foot and ankle-specific questions. The score covers different constructs that are not reported separately in subscales: pain, function including limitations of function, and other symptoms. Each of the 12 multiple-choice questions is scored from 0 to 4; thus, 0 total points represents the most severe disability and 48 represents normal function. The New Zealand National Joint Registry adopted this new scoring system in 2007, as did the Swedish Ankle Registry.

When the Swedish version of the questionnaire was created, it was translated into Swedish according to a standardized cross-cultural adaption procedure (Guillemin et al. 1993). The English questionnaire was forward translated into Swedish by 2 independent medically educated, native Swedish speakers. The Swedish version was then backward translated into English by a native English-speaking professional translator with no knowledge of the original version. The original English version was then compared with the backward-translated version, and if there was any discrepancy, these questions were made clearer by the 2 Swedish translators in a final Swedish version. This final version was then given to 10 patients with different hindfoot disabilities. They were asked to complete the questionnaire and were also asked if they understood all the questions. None of the volunteers reported any difficulty in completing the questionnaire. Regarding 8 of the questions in the original score, we had to make an important change in the text. Thus, in order to make it possible to use the questionnaire preoperatively and after procedures other than ankle replacement, “the ankle operated on” had to be changed to the Swedish equivalent of “the ankle in question”.

We adopted the following approach in cases of incompletely answered questionnaires in the SEFAS: (1) when results from 2 or more boxes were missing, the questionnaire was disregarded; (2) when the result from 1 box was missing, the mean result of the remaining 11 boxes was used; (3) when the patients gave 2 answers for 1 question, the worse outcome was recorded; and (4) when the patients had put a mark between 2 boxes, the worse outcome was recorded.

The New Zealand total ankle questionnaire can be found at www.cdhb.govt.nz/NJR and the self-reported foot and ankle questionnaire (SEFAS) in Swedish and English can be found at www.swedankle.se.

### The foot and ankle outcome score (FAOS)

The FAOS score is calculated from a patient-administrated 42-item questionnaire developed for foot and ankle-related disability (Roos et al. 2001). The FAOS covers 5 dimensions that are reported separately: (1) pain, (2) other symptoms, (3) activities of daily living, (4) function in sport and recreation, and (5) ankle-related quality of life (QoL). Standardized answer options are provided, and each question is rated on a scale from 0 to 4. A score is calculated for each subscale after which raw scores for each subscale are transformed to a scale ranging from 0 to 100 and presented graphically as the FAOS profile. The minimum possible total score for each subscale is 0 points, a condition that represents the most severe disability, whereas the maximum of 100 points represents normal ankle function. Missing data were assessed according to the user’s guide for FAOS (2003) (www.koos.nu).

### The SF-36 score

SF-36 is a score that is calculated from a validated generic questionnaire that contains 36 items (Sullivan et al. 1995, Patel et al. 2007). The score was developed for measuring health-related quality of life and is not especially related to the disease under consideration. The score is widely used when evaluating patients with different diseases, including musculoskeletal disorders. SF-36 measures 8 different dimensions of health. The minimum possible total score of SF-36 is 0 points, which represents the most severe disability, whereas 100 points represents the best possible health status. Finally, from the 8-dimension scores, 2 summary scales are calculated: 1 for physical health and 1 for mental health.

### The EuroQol (EQ-5D) score

The EQ-5D score is calculated from a self-administered questionnaire developed for measuring health outcome, and like the SF-36 score, does not specifically address foot and ankle disability (EuroQol Group 1990, Dolan 1997). The score is applicable to a wide range of health conditions and treatments, and is specifically designed as a complement to other quality-of-life measures such as the SF-36 (Schweikert et al. 2006). The questionnaire covers 5 different dimensions. The minimum possible total score of EQ-5D is 0.0 points, a condition that represents the most severe disability. EQ-5D also includes a visual analog scale (VAS) that assesses the general health state, ranging from 0 to 100. The worst possible health state is 0 and the best possible health state is 100.

### Patients

All questionnaires described above were sent to 135 patients registered in the Swedish Ankle Registry due to planned and/or accomplished replacement or fusion of the ankle joint. This included 74 women and 61 men with a median age of 63 years (26–85), during the period February 2008 to January 2010. Primary total ankle replacement (TAR) was planned and/or performed in 101 patients, total ankle revision in 9, and primary ankle fusion in 25. The index diagnosis was rheumatoid arthritis in 27 cases, idiopathic or posttraumatic osteoarthritis in 90 cases, and “miscellaneous” in 18 cases.

Informed written consent was obtained from the participants. The ethics committee of Lund University, Sweden approved the study (2009/698) and it was performed in line with the Helsinki Declaration.

### Evaluation of the scores

*Validity (n = 135). *Validity is an estimate of how well a score actually measures what it is supposed to measure. Criterion validity compares a new score with a gold standard but this was not applicable in our evaluation, as there is no gold standard for evaluation of foot and ankle disability. Construct validity concerns the extent to which a score relates to other scores consistent with theoretically derived hypotheses (de Groot et al. 2008). In the absence of a gold standard, the validity in our study was expressed in terms of construct validity, calculated with the Spearman’s correlation coefficient. For the validity test we compared the SEFAS with FAOS, SF-36 and EQ-5D. We took account of the fact that pain and function are the two most important symptoms for the patients and that pain and function therefore are the constructs of interest in each score. For convergent validity, we formulated 5 hypotheses. The correlation between SEFAS and FAOS subscales pain, activities of daily living (ADL), and symptoms, for SEFAS and SF-36 BP and PF should be ≥ 0.60. We also hypothesized that that SEFAS would show stronger correlation with FAOS pain and ADL than with SF-36 BP and PF. We formulated 3 hypotheses concerned discriminant validity: that the correlation between SEFAS and SF-36 GH, SF-36 RP, and the summary scale in SF-36 mental health should be ≤ 0.30. We hypothesized that all the other correlations between SEFAS and SF-36, the EQ-5D, and FAOS sport and recreation and quality of life should be between 0.30 and 0.60. For evaluation of the construct of interest, i.e. the pain and function in SEFAS, we related the pain-specific and function-specific questions separately to specific subscales in the other scores.

*Floor and ceiling effects (n = 135).* Floor and ceiling effects show the proportion of individuals who achieve the highest or lowest possible numeric value of a score and are considered present when more than 15% of the individuals achieve these values. Floor and ceiling effects can be used when evaluating content validity. A high floor and ceiling effect could make it difficult to distinguish patients from each other and also to measure changes in patients after intervention (Terwee et al. 2007, Wamper et al. 2010).

*Reliability (n = 62)*. Reliability is an estimate of the reproducibility of a score, and can be measured in different ways. In this study we evaluated test-retest reliability i.e. how well a score produces the same outcome when the questionnaire is given to the same individual on separate occasions but close to each other in time. For this evaluation, 78 consecutive patients were asked to answer and the questionnaires were sent to them twice, about 6 months after surgery, by post with a postage-paid return envelope. The second questionnaire was sent as soon as they had returned the first one. A maximum of 31 days was allowed to elapse between the dates of response, and the median time was 10 days. In the second round, 3 questionnaires were incompletely filled in, 4 were returned more than 4 weeks after the first questionnaire was returned, and 9 were not returned at all. This left 62 patients (40 women) with a median age of 64(26–85) years to be included in the reliability testing. We used intraclass correlation coefficient (ICC) with a two-way mixed model to evaluate test-retest reliability. The ICC is considered to be good at 0.70 and above (Streiner and Norman 2008). However, reliability is sometimes also reported from a wider perspective, to include internal consistency as an estimate of the extent to which the specific questions within a score are correlated to each other and therefore measure the same thing. When we evaluated reliability as internal consistency, we used the first questionnaire that was answered by the 62 patients described above. To test internal consistency, we used Cronbach’s α (CA).We used the widely accepted cutoff for CA at 0.70 and considered it to be good when it was 0.70 or higher (Streiner and Norman 2008)

*Agreement (n = 62).* Agreement is an estimate of the measurement error of a score. When we evaluated agreement, we used the 2 sets of questionnaires from the 62 patients described above and prepared the data as Bland-Altman plots (Bland and Altman 1986, Button and Pinney 2004). These plots show the difference between the SEFAS scores in the 2 questionnaires answered by the same patient (Bland and Altman 1986). Intraindividual variability of the functional measures was expressed as standard error of a single determination (S_method_), and is shown together with the coefficient of variation (in%) for all the scores in Table 1. The formula used was S_method_ = √(∑d_i_^2^/(2n)), where d_i_ is the difference between the ith paired measurement and n is the number of differences (Dahlberg 1940).

**Table 1.** Validity, reliability, and measurement errors of the different scores. Correlation analyses comparing SEFAS and the other scores. Data are presented as mean with 95% CI or standard deviation (SD), and as proportions (%)

	Validity		Reliability				Agreement
Questionnaire	Spearman Rho – SEFAS versus (95% CI)	Floor and ceiling effects (%)	Test mean (SD)	Retest mean (SD)	Intraclass correlation coefficient (ICC) (95%CI)	Cronbach’s α	S_method_
Number	135	135	62	62	62	62	62
SEFAS	1	0	29 (9.6)	29 (9.9)	0.92 (0.87–0.95)	0.96	2.7 (15%)
FAOS			–	–	–	–	
Pain	0.82 (0.76–0.88)	4.4	71 (21)	69 (22)	0.89 (0.82–0.93)	0.94	7.4 (11%)
Symptom	0.50 (0.37–0.63)	0	60 (16)	62 (15)	0.84 (0.75–0.90)	0.92	6.1 (10%)
ADL	0.77 (0.70–0.83)	1.5	77 (19)	77 (19)	0.96 (0.94–0.98)	0.98	3.7 (5%)
Sport/Recreation	0.42 (0.27–0.56)	34	24 (26)	24 (25)	0.78 (0.66–0.86)	0.88	12 (49%)
Quality of life	0.82 (0.76–0.88)	8.2	51 (25)	53 (25)	0.92 (0.87–0.95)	0.96	7.1 (14%)
EQ–5D	0.76 (0.68–0.83)	8.1	0.72 (0.22)	0.67 (0.24)	0.80 (0.68–0.87)	0.89	0.1 (16%)
Visual analog scale (VAS)	0.65 (0.53–0.75)	1.5	69 (19)	69 (20)	0.96 (0.94–0.98)	0.98	9.1 (13%)
SF–36			–	–	–	–	
Physical functioning (PF)	0.64 (0.53–0.74)	1.4	54 (23)	53 (23)	0.92 (0.88–0.95)	0.96	6.4 (12%)
Role limitations, physical (RP)	0.30 (0.14–0.46)	62	40 (43)	40 (43)	0.89 (0.82–0.93)	0.94	13 (32%)
Bodily pain (BP)	0.76 (0.68–0.83)	4.4	57 (24)	54 (23)	0.87 (0.79–0.92)	0.93	8.5 (15%)
General health (GH)	0.17 (0.00–0.34)	3	67 (22)	67 (23)	0.93 (0.89–0.96)	0.97	5.9 (9%)
Vitality (VT)	0.46 (0.31–0.59)	1.5	61 (21)	61 (24)	0.88 (0.80–0.92)	0.93	8.0 (13%)
Social functioning (SF)	0.42 (0.28–0.57)	33	82 (23)	82 (22)	0.72 (0.57–0.82)	0.84	12 (14%)
Role limitation, emotional (RE)	0.31 (0.15–0.46)	76	68 (43)	69 (42)	0.76 (0.64–0.85)	0.87	17 (25%)
Mental health (MH)	0.38 (0.22–0.52)	8.9	80 (17)	81 (16)	0.83 (0.74–0.90)	0.91	6.1 (12%)
Physical	0.51 (0.37–0.64)	–	38 (11)	37 (11)	0.88 (0.80–0.92)	0.93	3.8 (10%)
Mental	0.30 (0.13–0.45)	–	53 (14)	54 (13)	0.77 (0.64–0.86)	0.87	6.4 (12%)

*Responsiveness (n = 37). *Responsiveness is an estimate of how well a questionnaire detects changes over time or changes due to an intervention. When we evaluated responsiveness, we included 37 patients (22 women) with a median age of 65 (24–80) years who had answered the questionnaires just before and median 6 months (5–7) after replacement or fusion of their ankle. Only 20 of the 37 patients had completed the FAOS twice, due to the fact that this questionnaire was removed from Swedish Ankle Register in 2011. To test responsiveness, we used effect size (ES) and standardized response mean (SRM). Effect size is calculated by taking the difference between the means before and after treatment and dividing it by the standard deviation of the same measure before treatment. Cohen defined an effect size of 0.20 as small, one of 0.50 as moderate, and of 0.80 or greater as large (Cohen 1978). Standard response mean is calculated by taking the difference between the means before and after treatment and dividing it by the standard deviation of the change. SRM values are generally lower than the corresponding ES values (Liang 1995).

### Statistics

Statistical calculations were performed with SPSS software version 17.0. The statistics related to validity, reproducibility, reliability, agreement and responsiveness are described under each paragraph above. We calculated the confidence interval for the correlations according to Fisher’s z-transformation.

## Results

The construct validity analyses, including the Spearman correlation coefficients, are presented in Table 1. SEFAS mainly measures pain and function, and as expected we found the highest correlations between SEFAS and the subscales in FAOS and SF-36 that measure similar constructs. 70% or more of our predefined hypotheses could be confirmed. We also found higher correlations with FAOS pain and ADL than with SF-36 BP and PF, as expected. Concerning discriminant validity, the correlation between SEFAS and SF-36 GH, SF-36 RP, and the summary scale in SF-36 mental health were low. The correlations coefficients between the pain-specific questions in SEFAS and the FAOS subscale pain and SF-36 BP were 0.81 and 0.75, respectively. The correlation coefficients between the function-specific questions in SEFAS and FAOS subscale ADL and SF-36 BF were 0.68 and 0.50, respectively.

The content validity analysis, including floor and ceiling effects, is presented in Table 1. None of the patients had the highest possible or the lowest possible numeric value in the SEFAS; i.e., there was no floor or ceiling effect. The reliability analysis, including the test-retest and the interclass correlation coefficient (ICC), is also presented in Table 1. The ICC for SEFAS was 0.92 (95% CI: 0.88–0.95) and the Cronbach’s α was 0.96.

The agreement analysis (including Bland-Altman plots) is shown in the Figure. The measurement error analyses with S_method_ and the coefficient of variation for all the scores are given in Table 1.

**Figure:**
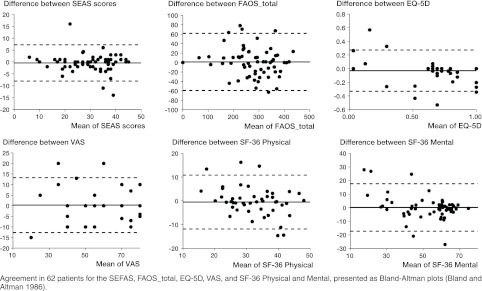
Agreement in 62 patients for the SEFAS, FAOS_total, EQ-5D, VAS, and SF-36 Physical and Mental, presented as Bland-Altman plots (Bland and Altman 1986).

The responsiveness analysis, including effect size (ES) and standardized response mean (SRM), is presented in Table 2. The ES for SEFAS was 1.44 and the SRM was 1.00.

**Table 2.** Responsiveness expressed as effect size (ES) and standard response mean (SRM) calculated for 37 patients before and 6 months after ankle arthroplasty operation. Due to missing answers, numbers are also shown

	n	Preoperatively (mean)	Postoperatively (mean)	Effect size (ES)	Standard response mean (SRM)
SEFAS	35	17	27	1.44	1
FAOS					
Pain	19	43	68	1.78	0.94
Symptom	20	46	52	0.47	0.55
ADL	19	53	73	1.36	0.89
Sport/Recreation	20	17	24	0.37	0.26
Quality of life	20	27	48	1.38	0.79
EQ-5D	36	0.4	0.6	0.93	0.81
VAS	29	50	67	0.65	0.53
SF-36					
Physical functioning (PF)	36	36	50	0.67	0.6
Role limitation, physical (RP)	35	28	36	0.23	0.15
Bodily pain (BP)	34	30	54	1.25	0.68
General health (GH)	33	70	69	–0.04	–0.06
Vitality (VT)	34	53	57	0.14	0.13
Social functioning (SF)	33	75	82	0.31	0.28
Role limitation, emotional (RE)	32	53	72	0.44	0.33
Mental health (MH)	34	79	80	0.07	0.08
Physical	30	31	36	0.66	0.49
Mental	30	51	54	0.26	0.23

## Discussion

This study shows that the SEFAS self-reported foot and ankle score has good validity, reliability, and responsiveness, which could be used to evaluate osteoarthritis or inflammatory arthritis of the ankle both before and after surgical intervention. For evaluation of overall validity of an outcome instrument, several clinimetric properties should be of sufficient quality (Bremander et al. 2003, Terwee et al. 2007). We found that these properties of the SEFAS were comparable with those of the ankle specific-score FAOS. The FAOS is a widely used foot and ankle-specific score that has been translated to several languages (Goksel Karatepe et al. 2009), but to our knowledge has only been validated for ankle ligament reconstructions (Roos et al. 2001). The correlation between SEFAS and the FAOS subscale for sport and recreation was low, as the FAOS may better capture sports-specific deficits while the SEFAS reflects everyday activity. There was also lower correlation with the FAOS symptoms subscale, which includes various unspecific phenomena.

In contrast to SEFAS and FAOS, SF-36 is a generic instrument that measures health-related quality of life. This instrument is widely used and validated for outcome assessment in a variety of general diseases, and is therefore often used in the process of validation of new scores. As expected we found both convergent and divergent validity when comparing the ankle-specific SEFAS with this generic SF-36.

EQ-5D is another generic score used in numerous orthopedic and other studies, and for various indications. In the present study we found a high correlation between the ankle-specific SEFAS and EQ-5D, reflecting the fact that osteoarthritis and inflammatory arthritis in the ankle have an effect on quality of life.

The foot function index (FFI) is another validated, self-reported foot questionnaire that was originally validated in patients with rheumatoid arthritis (Budiman-Mak et al. 1991), but it was revised and there are now several versions in different languages. A number of problems have been apparent with the FFI score (Trevethan 2010). There has also been a report inferring that this score is of less value in patients undergoing ankle replacement (Naal et al. 2010).

The subjective visual-analog scale of the foot and ankle (VAS FA) is a recently reported and validated questionnaire that shows good correlation with the SF-36 (Richter et al. 2006). However, since the reliability, content validity, and responsiveness of the VAS FA have (to our knowledge) never been evaluated, this ought to be done before the instrument is introduced for the evaluation of ankle disability. None of the above-mentioned scores have been translated into Swedish.

In addition to the scores evaluated in this report, there are clinician-based ankle-specific scores. The difference between self-reported or patient-reported outcome and clinician-based scores is basically the fact that the clinician-based score is dependent on anyone who examines the patient, and of course the assessment can be more or less subjective. The Kofoed and Mazur scores are 2 clinician-based scores that (to our knowledge) have never been validated (Button and Pinney 2004, Naal et al. 2010). The American orthopaedic foot and ankle score (AOFAS) (Kitaoka et al. 1994, Lau et al. 2005) is without doubt the most commonly used instrument, but it has the disadvantage of not only including patient-related information but also of requiring a professional clinical examination. In practice, this precludes this score and the above-mentioned scores from being used in large registry studies. The AOFAS has also been the subject of other criticism. SooHoo et al. (2003) reported poor construct validity when comparing AOFAS with SF-36 in patients with foot and ankle disability, lower than when SF-36 was compared with scores evaluating shoulder, knee, and upper extremity disability. Another concern is that clinician-based scores do not adequately take into account the patient’s point of view. These problems were summarized by Naal et al. (2010), who reported that self-reported outcome instruments allow a more complete estimation of the patient’s health status and of other issues relevant to the patient.

Floor and ceiling effects must also be evaluated when introducing new scores, as a ceiling effect makes it impossible to grade improvements after interventions. This estimate is most important when evaluating registry data for clinicians and healthcare politicians when allocating resources to specific interventions—as pointed out by Wamper et al. (2010) when discussing the Harris hip score. In contrast to the findings in the other instruments that we evaluated, SEFAS did not show floor or ceiling effects. Our study population was highly selected, consisting of patients with ankle osteoarthritis, which could be a reason for these results.

The test-retest reliability was good for all the questionnaires, with an ICC of > 0.70. The internal consistency was also good for all scores, with Cronbach’s α values above 0.70. However, some authors have pointed out that a value of Cronbach’s α that is too high may be a problem, indicating that different questions in the questionnaire capture the same symptom or deficits. In this respect, a Cronbach’s α of 0.96 in the SEFAS may be too high to be ideal (Terwee et al. 2007).

Reliability and agreement are both concepts concerning estimation of the reproducibility of different instruments (Terwee et al. 2007). Agreement includes estimation of the absolute measurement error, i.e. the deviation of one measurement from another, while reliability estimates how well different patients can be distinguished from each other, when taking the measurement error into account. The ICC and Cronbach’s α are the most frequently used parameters when estimating reliability, while a variety of parameters have been used when describing agreement (Streiner and Norman 2008). We used the Bland-Altman plot (Bland and Altman 1986, Terwee et al. 2007). Bland-Altman plot indicated that there is good agreement between responses to SEFAS questionnaires when answered more than once.

The responsiveness is an important consideration when estimating the effect of an intervention such as arthrodesis or arthroplasty of the ankle joint. In this study, the SEFAS questionnaire showed good responsiveness, calculated with both ES and SRM as we expected, but the sample size was somewhat low—which is a limitation. Also, the other region-specific score FAOS shows good responsiveness, which is one of the known advantages of region-specific scores.

The strengths of our study include the structural evaluation of a new self-administrated ankle-specific score against other commonly used scores (both foot and ankle-specific) and generic scores regarding reliability, validity, and responsiveness. One of the limitations of our study is that we did not include the satisfacion-rate of the patient. It would also have been advantageous if patients with different ankle diagnoses and foot disorders had been evaluated in the same manner as separate cohorts, as our inferences can now only be applied to patients with ankle arthritis and only to surgical intervention with arthroplasty or arthrodesis.

We conclude that SEFAS is a valuable self-administrated questionnaire for evaluating patients with osteoarthritis and inflammatory arthritis of the ankle and the outcome of ankle surgery. It could, for example, be a suitable tool for patient-related outcome measures (PROMs) in connection with national registries. Further studies ought to be conducted to determine whether SEFAS can also be used to estimate the function of patients with all kinds of foot and ankle disorders.
